# Supplementary figures and images for: Comprehensive discovery of CRISPR-targeted terminally redundant sequences in the human gut metagenome: Viruses, plasmids, and more
Source: PLoS Comput Biol. 2021 Oct 21;17(10):e1009428. doi: 10.1371/journal.pcbi.1009428 (PMC8530359; doi:10.1371/journal.pcbi.1009428)

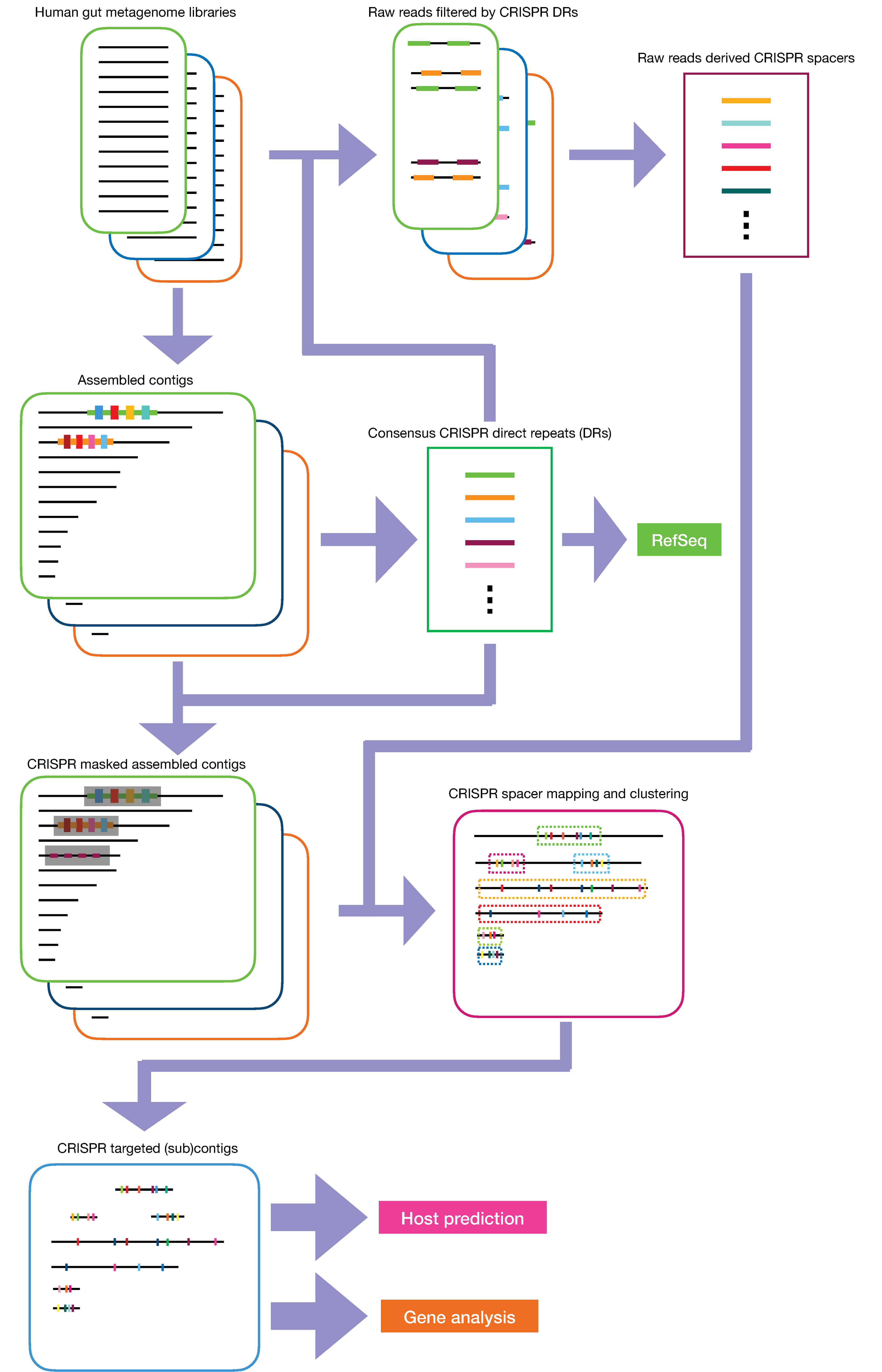

Supplement: S1 Fig — Human gut metagenome libraries were preprocessed to remove adapters, phi X, and human sequences. After correcting sequencing errors, libraries were assembled. Clustered regularly interspaced short palindromic repeats (CRISPR) loci were discovered from the assembled contigs. Consensus direct repeats (DRs) from the discovered CRISPR loci were used to extract spacers, mask the CRISPR loci, and predict the host. All unique CRISPR spacers were mapped to contigs to discover the protospacer loci. Spacers were clustered based on the co-occurrence of the associated protospacers. Sequences targeted by more than 30% of the members of a spacer cluster were extracted and used for further analysis. (TIF) [file pcbi.1009428.s001.tif]

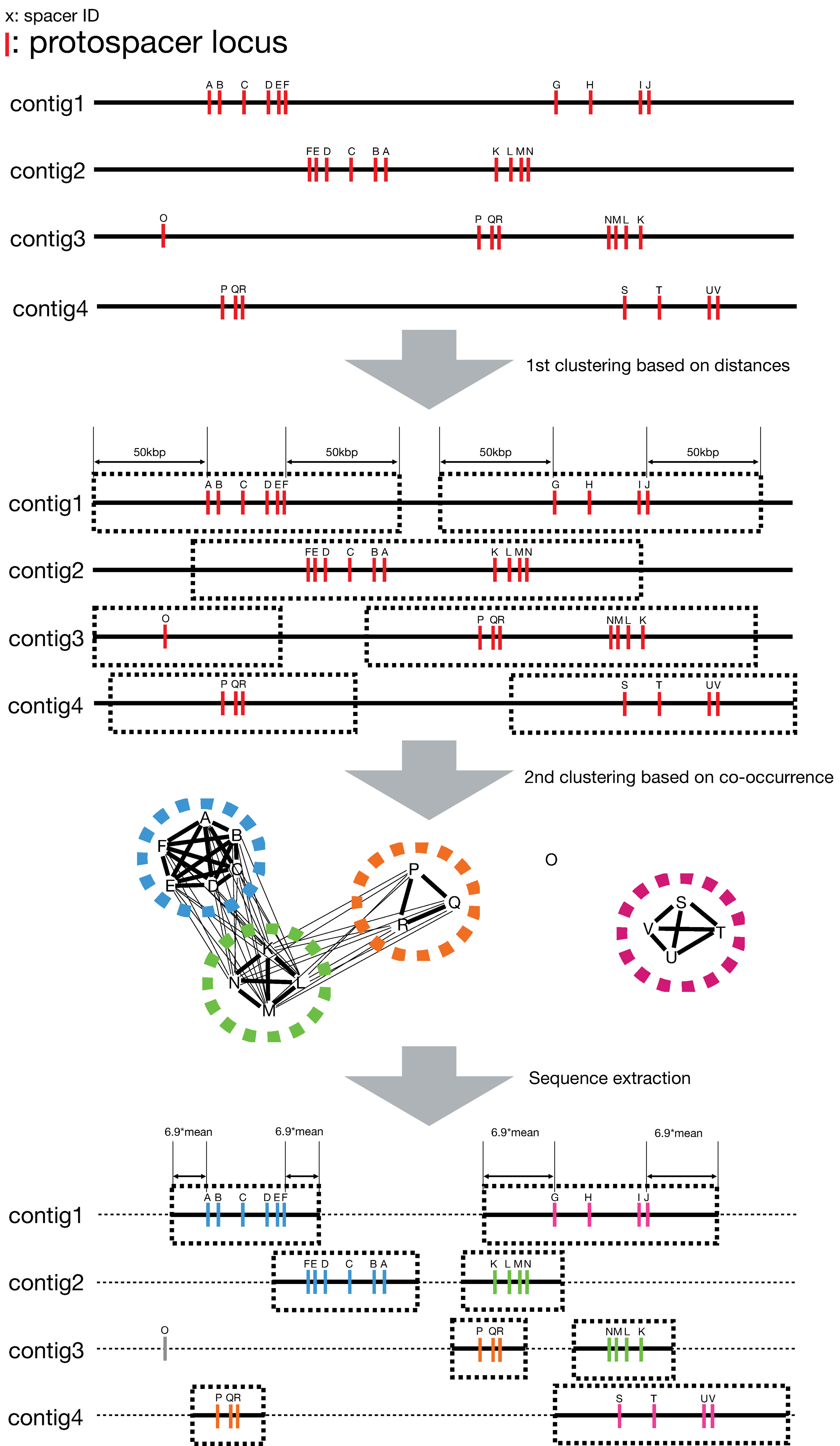

Supplement: S2 Fig — Initially, protospacer loci were clustered based on the distance between them. Within initial clusters, co-occurrences of protospacers were counted and used to construct an undirected graph. The nodes (spacers) in the undirected graph were further clustered using the Markov clustering algorithm. The mean distances between adjacent protospacer loci within clusters were calculated and used to extract CRISPR-targeted sequences. The length and number of protospacers shown here are conceptual and not based on observed data. (TIF) [file pcbi.1009428.s002.tif]

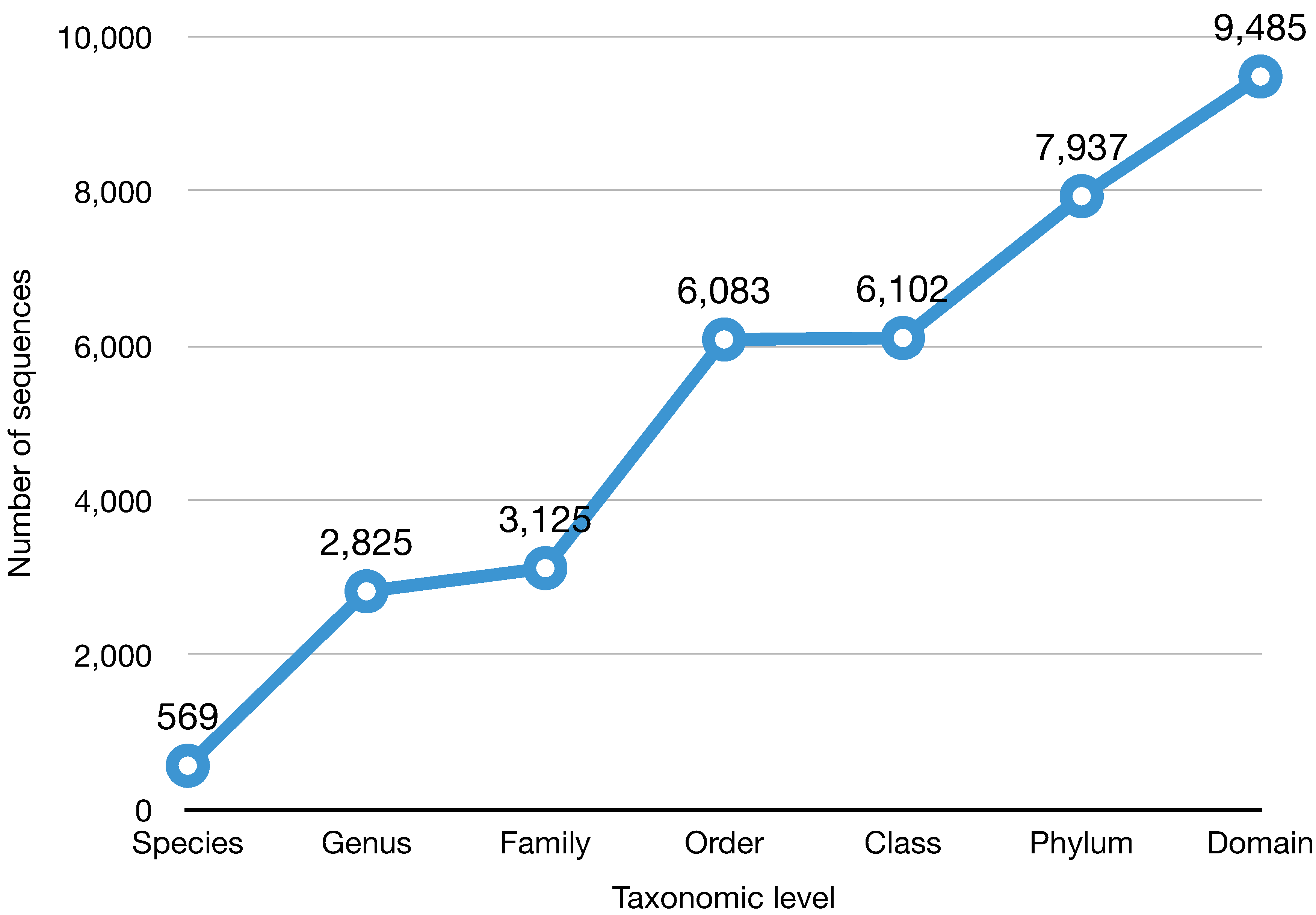

Supplement: S3 Fig — (TIF) [file pcbi.1009428.s003.tif]

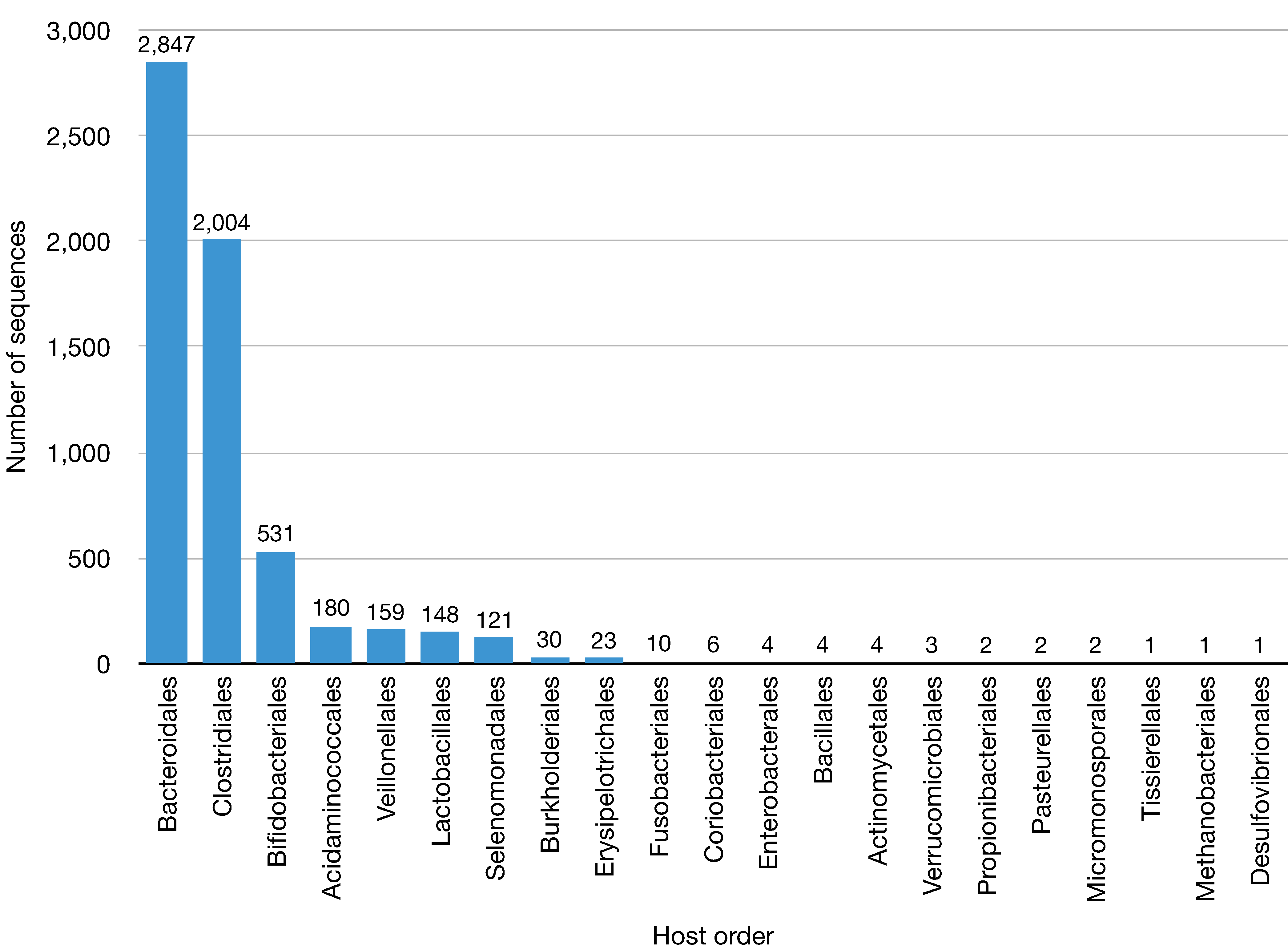

Supplement: S4 Fig — (TIF) [file pcbi.1009428.s004.tif]

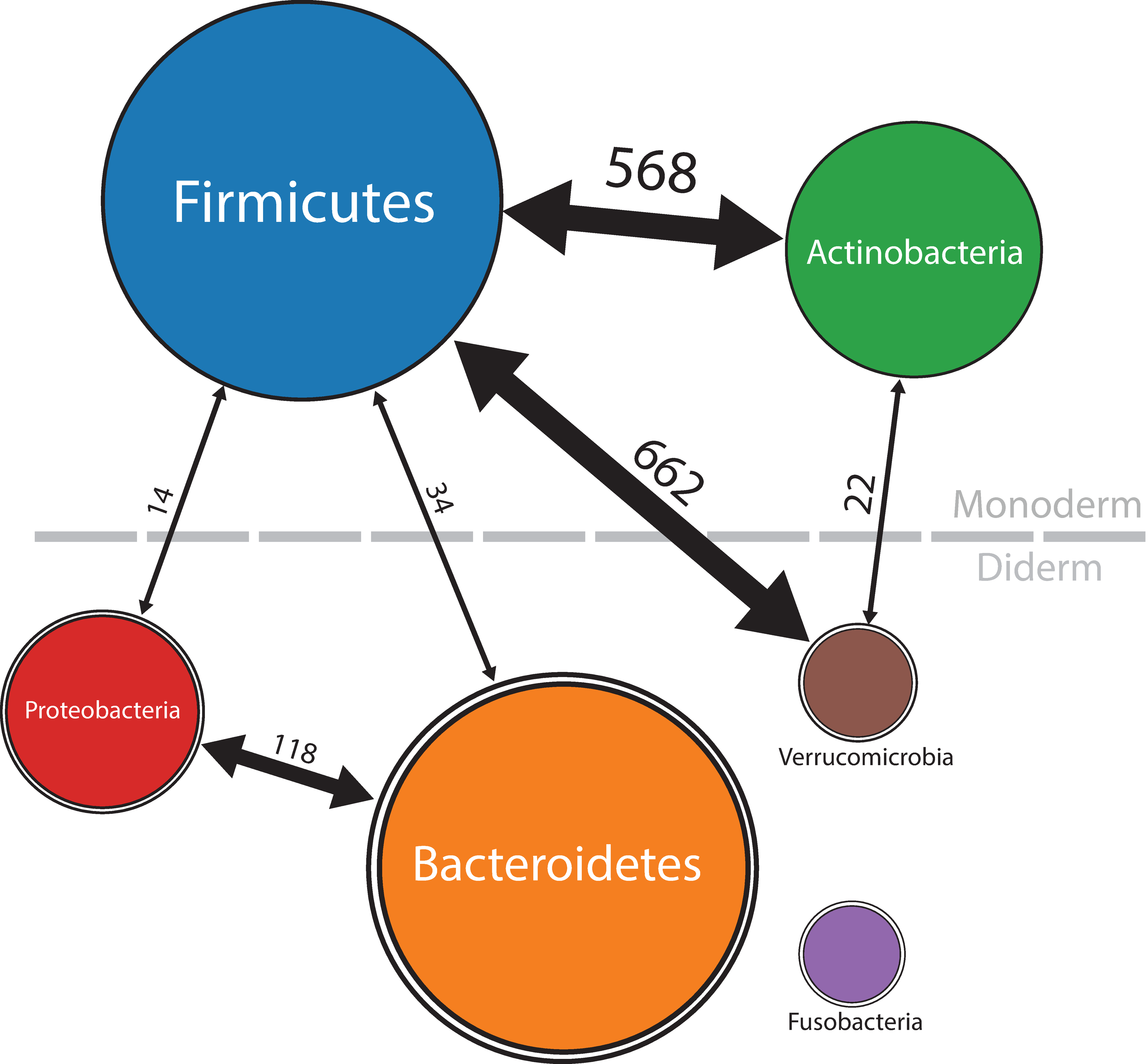

Supplement: S5 Fig — Circle size approximately represents the popularity of the respective host. The bidirectional arrows connect the top two phyla according to host-assigned protospacer counts (i.e., protospacers most often associated with CRISPR DRs are assigned to these two phyla). The numbers on the arrows are counts of the number of TR sequences associated with the connected phyla. (TIF) [file pcbi.1009428.s005.tif]

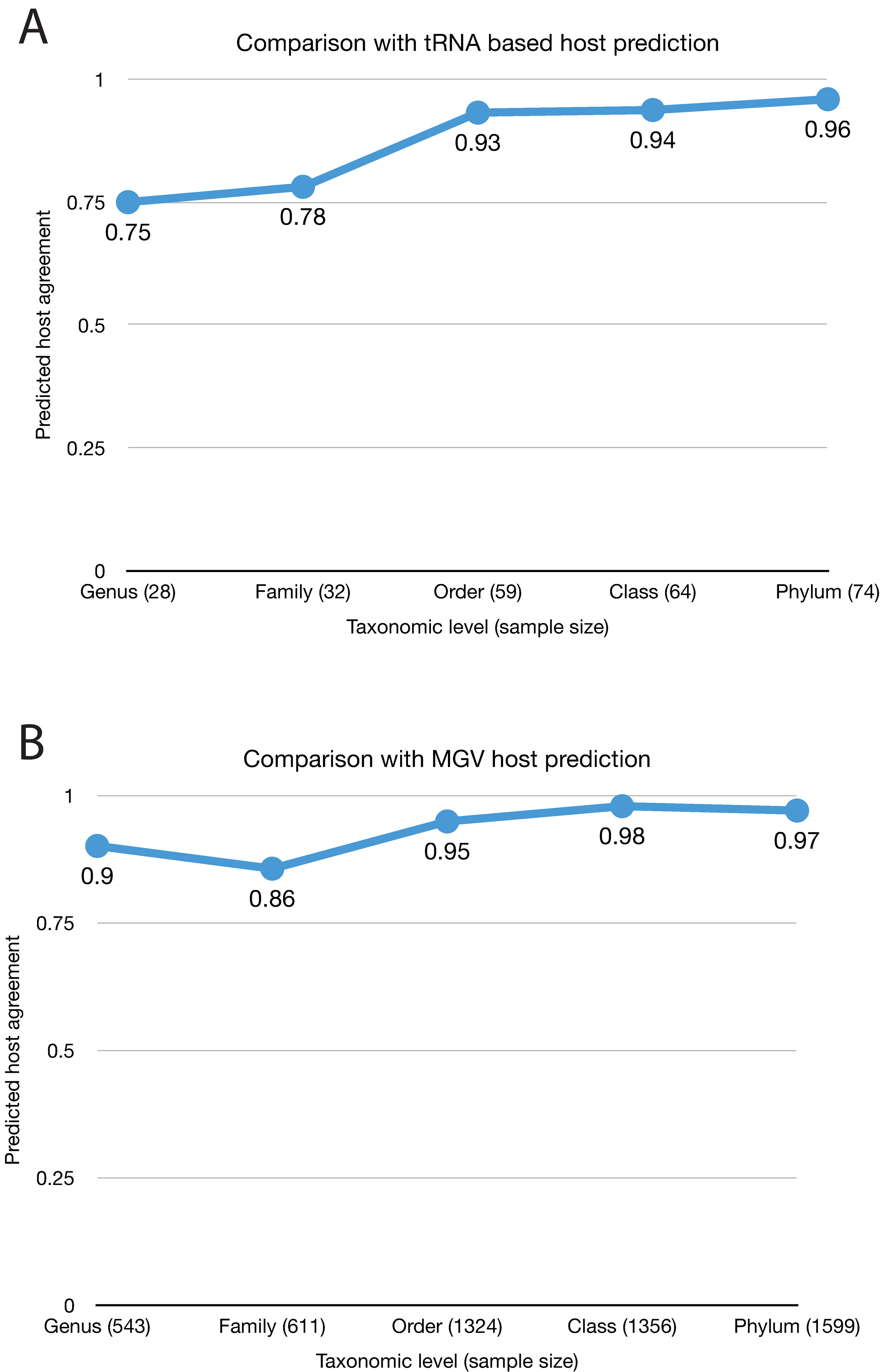

Supplement: S6 Fig — (A) Host prediction comparison between DR-based and tRNA-based methods. (B) Host prediction comparison between MGV and this study. (TIF) [file pcbi.1009428.s006.tif]

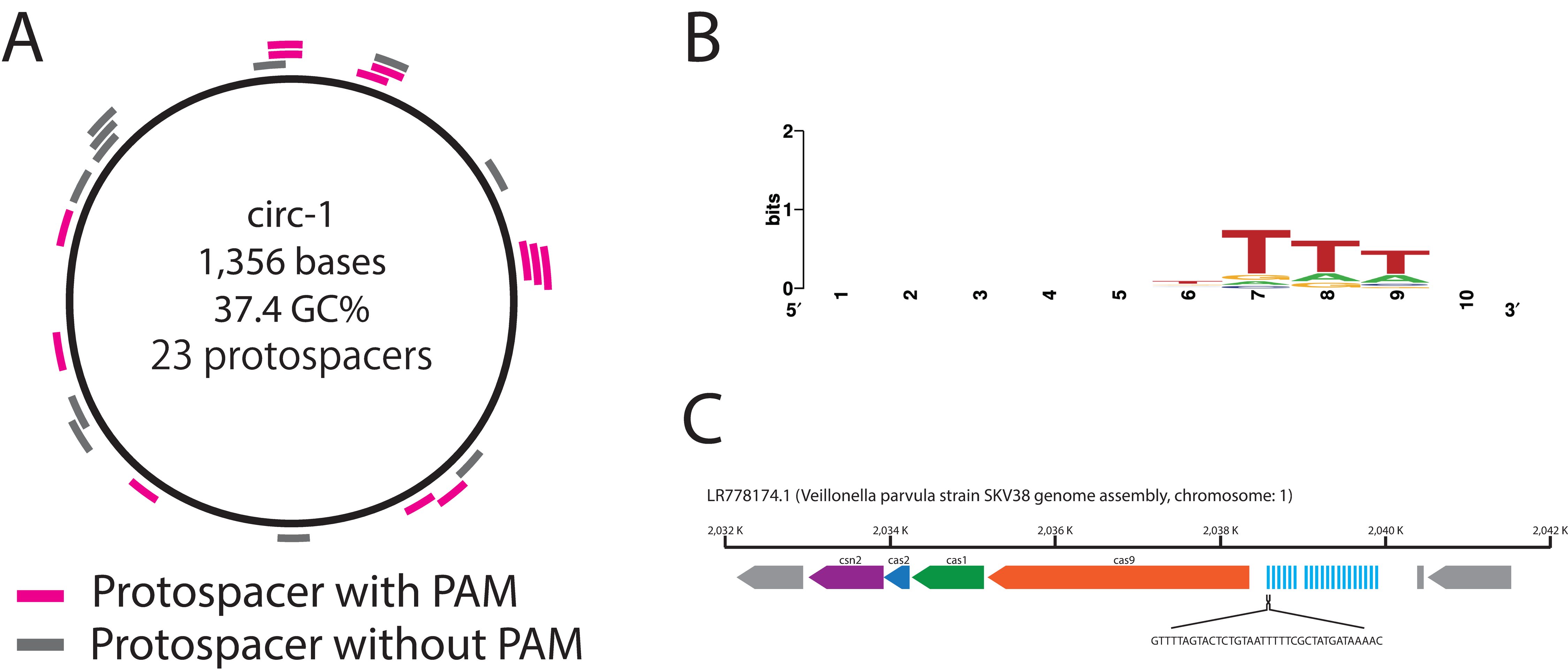

Supplement: S7 Fig — (A) Genomic map of circ-1. The circle represents the circular genome of circ-1. The positions of protospacers are indicated outside the circle. The protospacers with and without PAM were colored magenta and dark gray, respectively. (B) PAM of circ-1 protospacers. Both adjacent sequences of protospacer positions up to 10 bases were collected and then aligned, to generate a logo using WebLogo [78]. (C) DR-aligned locus of LR778174.1. The cyan bars are the DR-aligned positions. The genes related to the Class 2 Cas system were annotated using colors. (TIF) [file pcbi.1009428.s007.tif]

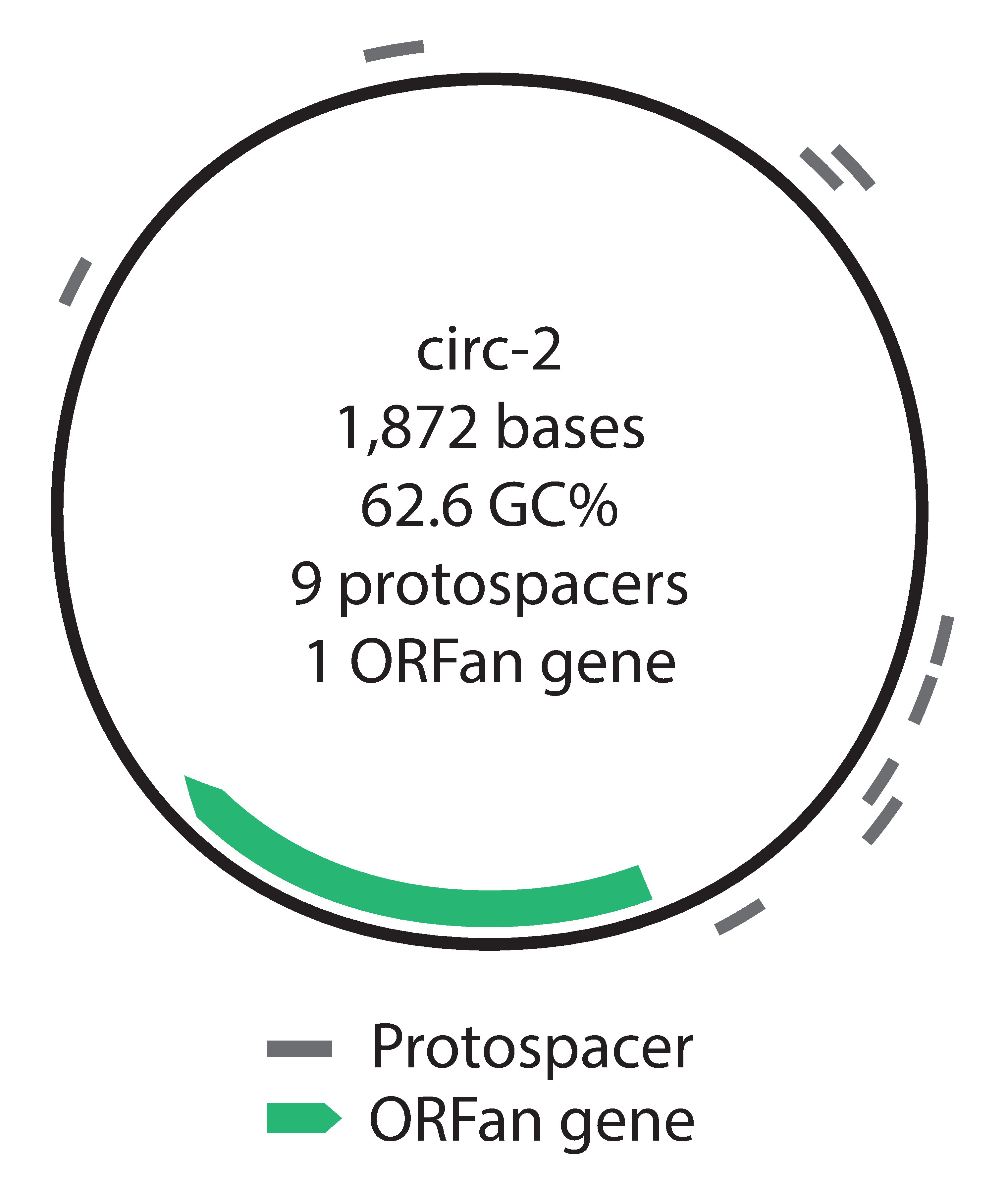

Supplement: S8 Fig — Genomic map of circ-2. The positions of protospacers and an ORFan gene are depicted outside and inside the circle, respectively. (TIF) [file pcbi.1009428.s008.tif]

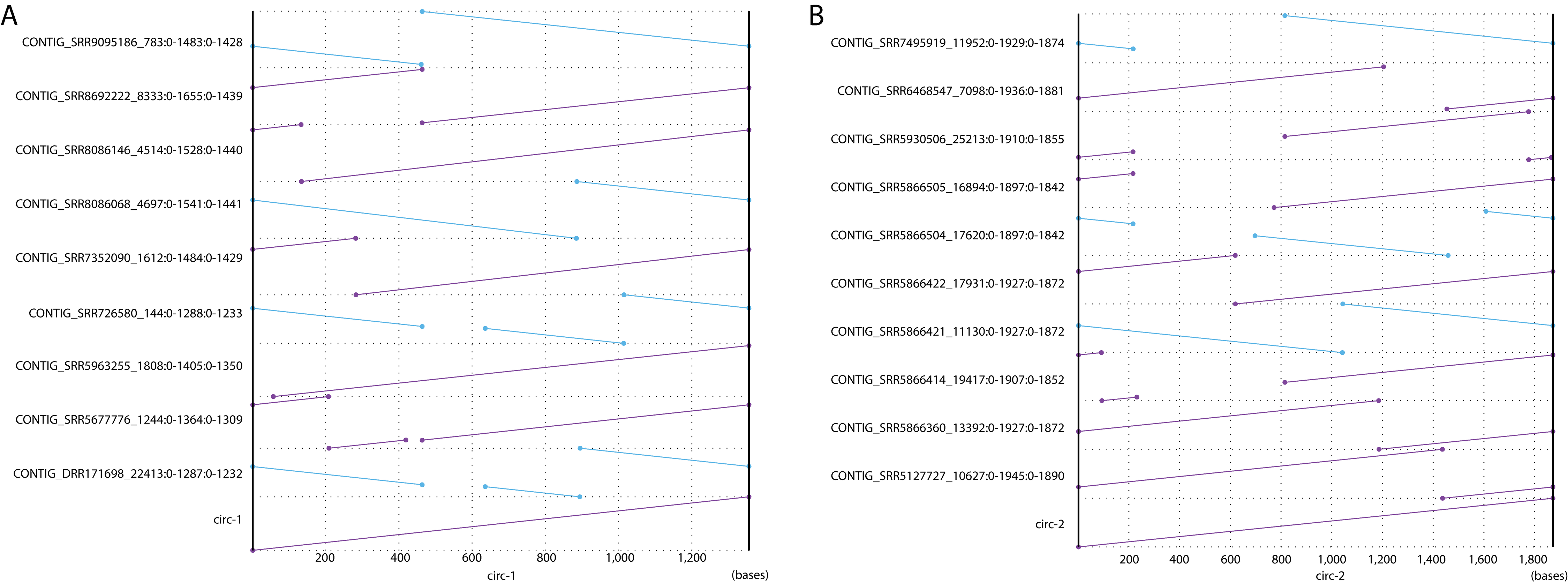

Supplement: S9 Fig — (A) circ-1 and (B) circ-2 dot plot representations of genome comparisons. The representative and similar genomes were aligned using nucmer [79], then plotted using mummerplot. For circ-1, the 10 most-similar genomes were selected. (TIF) [file pcbi.1009428.s009.tif]
