# Supplementary material for: Comprehensive discovery of CRISPR-targeted terminally redundant sequences in the human gut metagenome: Viruses, plasmids, and more
Source: PLoS Comput Biol. 2021 Oct 21;17(10):e1009428. doi: 10.1371/journal.pcbi.1009428 (PMC8530359; doi:10.1371/journal.pcbi.1009428)
Supplement: Attachment — Submitted filename: Response_to_reviewers.docx [file pcbi.1009428.s012.docx]

Responses to Reviewer Questions

Reviewer #1: Sugimoto et al present a census of viruses from the human gut by matching CRISPR spacers to sequences carrying protospacers. The approach is straightforward and timely, and the findings are, on the whole, credible and interesting. The multitude of identified small ssDNA viruses, the majority of these previously unknown, is notable. There are some concerns, though.

1. The cut-off used for protospacer detection, 93% identity, is not as stringent as the authors claim. This means 2 mismatches for the typical spacer length are allowed, which is not error-proof, so an assessment of the false positive rate is necessary.

Thank you for this critical point. Our purpose of using CRISPR spacers was to discover viral genomes targeted by CRISPR. These CRISPR targeted genomes are not necessarily being targeted by CRISPR at present, but mutated descendants escaped from CRISPR targetings, which is also our interest. To capture such escaped mutants in this study, we chose to use looser criteria (93%; telerate two mismatches) than the widely used criteria (95%; telerate one mismatch) in the previous studies. While the looser criteria may increase the sensitivity to capture the escaped mutants, the assessment of the spacer hit specificity was desired as the reviewer pointed out. To evaluate the specificity of spacer hit in a given criteria, we required “control” sequences; sequences not containing a genuine protospacer. However, there is a fundamental difficulty to design such control sequences, due to the nature of CRISPR spacers. It is known that CRISPR spacers could be acquired from various genomic sources, including DNA/RNA virus, plasmid, transposon and chromosomal DNA. Furthermore, partial prophage genomes are regularly encoded in bacterial/archaeal genomes. Therefore, we cannot define a metagenomic sequence that could absolutely not be a source of CRISPR spacer acquisition, making us unable to design a control. To complement this, we applied a graph-based clustering method to extract the regions with co-localized protospacers (S2 Fig), and evaluated the spacer hits by chance in a given criteria (Fig 5).

While the genuine protospacers from a viral genome are expected to co-localize together in a relatively small region, the false positive protospacers are expected to be scattered across the metagenome contigs almost randomly. We sought to extract the sequences with co-localized protospacers by using a graph-based clustering method (page 7, line 147). The aligned spacers were clustered based on the co-occurrence of protospacers. The clusters with less tightly connected (global clustering coefficient < 0.5), or small sizes (< 10) were rejected. 12,749 clusters comprising 591,189 spacers remained. These spacers are confidently co-aligned together with other spacers within the local regions. We considered a region containing at least 30% of a cluster member a genuine CRISPR targeted region. Such sequences were extracted and further analyzed.

The spacer hits-by-chance evaluation is analogous to the E-value from the blast program. This measurement was assessed as follows. With 95% identity, 192,801 spacers were aligned to terminally redundant (TR) sequences and one spacer was aligned to scrambled sequences. With 93% identity, 251,467 spacer sequences were aligned to TR sequences and 14 spacers were aligned to scrambled sequences (Fig 5). Although we observed an increased number of hits to scrambled sequences with 93% identity, 14 spacers would be acceptable considering that 58,666 more spacers were identified. Accordingly, we believe that the increased number of hits with the 93% identity threshold is unlikely to be the result of chance but more likely by the sequence diversity of TR sequences driven by selection pressures, including CRISPR targeting.

Overall, we conclude that the extracted CRISPR targeted sequences are mostly composed of genuine protospacers. We sought to evaluate the CRISPR avoidance phenomenon of viral genomes by using the discovered mutated genomes, which only briefly discussed in this manuscript (page 18, line 420 and page 22, line 516). We leave the further analysis of this topic in the future study.

2. The authors claim “may entirely novel genomes of unknown taxa”, but this claim is misleading because novelty here means failing an extremely stringent threshold, 85% nucleotide identity. It is disingenuous to claim “entire” novelty and actually any novelty as such based on this threshold. Roughly, this result means that a number of viruses could not be included in already know genera. All novelty statements have to be revised to this effect.

We agree that claiming novelty in the given identity threshold is misleading and did not provide information about the taxonomy from an evolutionary perspective. Since the purpose of our database comparison was validation and narrowing our results to previously uncharacterized genomes, all novelty statements were replaced with the phrase, “did not produce hits in the databases with our criteria.”

3. The “novel” genomes were then searched with capsid protein profiles to determine the fraction of the “novel” sequences that were likely to come from viruses. However, neither the selection of the capsid genes nor the matching criteria are adequately described. Only the cut-off score is indicated which in itself tell nothing about the specificity and sensitivity of the search.

As noted, we admit that the previous pipeline for discovering capsid protein was unnecessarily complicated and lacking in sensitivity and specificity measures. The extant pipeline was based on only known capsid protein sequences. This procedure yields erroneous results because it ignores noncapsid sequences that could be aligned to the query with better scores. To simplify the pipeline and treat all database sequences equally, we completely redesigned the gene annotation pipeline and included capsid detection. Briefly, we used all representative amino acid sequences as seeds for jackhmmer iterations and used for building HMMs as the query for HHsearch in PDB. The hit criteria were set to 80% probability with a <1e-3 E-value (page 8, line 157). The new pipeline is simpler and straightforward, and detection of capsid genes is no longer a separate analysis. With the new pipeline, the noise you would previously see in the clustering heatmap disappeared, indicating the increased specificity of our method, as we describe in the next paragraph. Also, the detected capsid genes are annotated based on their types: HK97, Microviridae MCP, and Inoviridae MCP. This information helps to infer the viral taxonomy.

The sensitivity and specificity of capsid detection were assessed by inspecting the annotation result of the TR sequences similar to RefSeq-recorded virus and plasmid genomes. Among 588 RefSeq virus recorded TR sequences, we detected capsid genes from 577 TR sequences (271 HK97 capsids, 306 Microviridae MCPs, and 0 Inoviridae MCP) (98.13%). On the other hand, among 1096 RefSeq plasmid recorded TR sequences, we detected capsid genes from 6 TR sequences (6 HK97 capsids) (0.55%). Our pipeline successfully detected capsids from most reference viral genomes and almost none from non-viral genomes. Accordingly, we conclude that our pipeline has acceptable sensitivity and specificity. This issue is described in the revised text (page 26, line 616).

4. There is effectively no attempt to analyze the genomes of “novel” viruses and place them within or outside the current virus classification. To this reviewer, this is a major deficiency. We want to know whether the approach implemented in this paper actually allows one to discover truly novel viruses, those without close evolutionary connections to known families. The current version of the manuscript does not answer this question.

We have taken this comment with significant consideration, particularly about the TR sequences without detectable capsids. We reinvestigated these sequences and found that the majority of them are likely of plasmid origin (page 9, line 179). Furthermore, we discovered that some small conserved contigs are likely noncoding. We were unable to conclude whether or not these are of viral origin, but we think the discovery is interesting and indicates that our method could enable the discovery of previously uncharacterized mobile genetic elements. Therefore, these topics were newly added to the revised manuscript (page 9, line 186).

Language: editing by a native English speaker is desirable - the manuscript is not written particularly poorly, overall, but nevertheless, there are quite a few obscure or ungrammatical sentences.

We submitted our manuscript for editing by a native speaker before the first submission, but probably it was not sufficient. We asked again for more precise and intense English editing for this submission.

Reviewer #2: The authors present a methodology to detect viral sequences and their microbial hosts based on CRISPR spacers retrieved from assembled contigs, as well as from unassembled read data. They used the suggested approach to perform an analysis of phage-host interactions in gut-associated microbiomes. The authors conclude by performing a phylogenetic analysis of diversity generating retroelement (DGRs) present in crAssphage, an abundant phage in gut microbial communities.

Despite the extensive bioinformatic analyses, I found fundamental flaws and little conceptual novelty in the presented research.

The methodology is based on using assemblies to associate microbial host cell with CRISPR directed repeats (DRs) and then seeking these DRs in raw read data to recruit large spacer databases associated with each host. The last step is using these spacers to detect protospacers, presumably in viruses, that match the spacers, thus linking hosts to viruses. The problem with this linkage of host-DR-spacer-virus approach is that it does not provide a unique mapping from host to virus. Specifically, the host-DR link is problematic, as it has been demonstrated that evolutionary distant bacteria can contain perfectly identical DRs. This occurs due to the high rates of HGT these systems undergo. The consequence of identical DRs in a completely different host is that, unless there is evidence connecting relevant spacer-containing reads to a specific contig, the genuine microbial host cannot be inferred by the suggested process. This is evident even from the results presented by the authors themselves, who state they observe high rates of phages that are reportedly targeted by bacteria from different phyla (“1,418 TR sequences (12.5%) were predicted to be targeted by multiple phyla”). Without evidence to the contrary, the most likely explanation is that these 12.5% of the targets are not infecting bacteria from different phyla (including viruses allegedly infecting both Gram-positive and Gram-negative bacteria), but rather the CRISPR-Cas systems in these hosts cells have identical DRs. This phenomenon is expected to be even more severe in lower taxonomic levels, which more readily exchange genetic material, rendering the host-virus association not informative.

We appreciate this critical point of the host-DR connection problem related to horizontal transfer of the CRISPR-Cas system. In the first submission, we were aware of this phenomenon and therefore did not taxonomically assign DR shared between different taxa in a given taxonomic level. Such DRs do not contribute to the targeting host prediction. However, our results showing viruses infecting both Gram-positive and Gram-negative bacteria are highly suspicious and therefore required further investigation. Through manual inspection of one of these TR sequences, we found that the sequence was targeted by the protospacers associated with two similar DRs (82.9% identity). Although this identity value is sufficiently low for each DR to be taxonomically assigned to *Verrucomicrobia* (*Akkermansia muciniphila*) and *Firmicutes (Clostridia)*, we found two phage-related genes adjacent to the DR-aligned locus of *Akkermansia muciniphila*, suggesting the CRISPR-Cas system in *Akkermansia muciniphila* was introduced by recent phage integration. In addition, we searched the TR sequence from the putative hosts to find a signature of horizontal gene transfer from the putative virus to the genuine hosts. While the TR sequence was partially aligned to a *Clostridia* species genome, no significant hit was found from *Akkermansia muciniphila*. Accordingly, there was no strong evidence to support that the putative virus infects both phyla at present. This result was added to the manuscript (page 11, line 252), and the term “multiple” was replaced with “ambiguous” for clarity.

Further, in the title and along the manuscript, the authors suggest that viral sequences can be detected based on CRISPR-matching protospacers. This is misleading, as it is known (and indicated by the authors at some point) that CRISPR spacers do not target viruses exclusively. Retrieved sequences can include plasmid, transposable elements, and even microbial chromosomes. While not abundant, self-targeting spacers have been found and are explained by silencing of CRISPR-Cas systems using different mechanisms, such as anti-CRISPRs or mutations in the flanking repeats.

The target analysis is based on retrieved terminally redundant sequences. The authors state that they have “successfully extracted 11,391 terminally redundant (TR) CRISPR-targeted sequences ranging from 894 to 292,414 bases. These sequences are expected to be complete or near-complete circular genomes”. I find this assumption to be very problematic. The authors do try to substantiate their assumption by searching for phage signature genes in TR sequences and report finding them mostly in large (>20kb) contigs. For short TRs, they mention that they “are unable to strongly conclude whether the remaining small TR sequences are viruses or not at this point.” Still, throughout the manuscript, they treat those TRs as complete genomes or plasmids. Terminally redundant sequences, especially ones as short as 894 bp, could have other origins, which the authors should have considered. Transposons, for example, can be difficult for assembly algorithms to include within contigs and can have terminal repeats, appearing as TR sequences.

We appreciated the reviewer’s comment and have taken it with significant consideration, particularly about the TR sequences without detectable capsids. First, we compared the TR sequences against the known transposon database (ICEberg) and detected that nine sequences are highly similar to the database recorded sequence (page 16, line 362). Next, we scrutinized the remaining sequences without detectable capsids, and we found that majority of them are likely plasmid origin based on the presence of plasmid-related genes such as ParA (page 9, line 179). Furthermore, we discovered that 95 small likely noncoding circular genomes from multiple samples. We were unable to conclude that these are viral origin at this point. However, the discovery might be of interest. Accordingly, these topics were added to the manuscript (page 9, line 186).

A key component in the suggested approach is the detection of spacers in read data. Yet, the authors failed to mention numerous previous studies applying this approach, including tools designed specifically to perform this task, such as CRASS (Skennerton et al., NAR 2013) and MetaCRAST (Moller et al., PeerJ 2017).

The presented articles are now cited in the manuscript (page 6, line 120).

Finally, the concluding evolutionary analysis of DGRs in crAssphage seems unrelated to the rest of the manuscript.

The DGRs in the crAssphages section were intended to show the discussion related to evolution using discovered genomes. We agree with the reviewers’ comment indicating that it diverges somewhat from the article’s purpose. We decided to remove the entire section about DGRs and preserved it for future study.

Reviewer #3: Sugimoto and colleagues present a de novo virus detection pipeline based on using CRISPR spacers, and apply this approach to over 11,000 human gut metagenomes to detect viral genomes and infer a surprising 70% with a putative host prediction. The authors additionally focus analyses on diversity-generating retroelement (DGR) loci from the widely distributed crAssphage and surmise that there is a common ancestor with human- and baboon-derived sequences. Overall, for a new virus detection pipeline, I would have expected a thorough benchmarking of the methods compared to existing methods (eg, VirSorter or VirFinder) and detailed quality assessment based on the recent MIUViGs standards (Roux et al, Nature Biotech 2019). As written, the pipeline and resultant data are very descriptive and lack a statistically robust assessment of false/true positive recovery rates. Further, it is unclear the level of novelty of this work compared to other approaches to detect uncultivated viruses from metagenomic data. For example, the authors compare the recovery rate of TR sequences >20Kb and nearly all are recovered in existing databases (IMG/VR, RefSeq Plasmid, GVD), with the remaining <20Kb not well represented in databases, but also questionable as to whether they even represent viral sequences. Below the authors should consider several major concerns with the current manuscript, and seek to address these to better validate their pipeline.

Thank you for providing the comments about validation of the pipeline.

We considered providing the MIUViG-based metadata during the early iterations of the analysis, however, we decided to leave this task in the future study. The purpose of this study is to establish the viral detection pipeline and investigate the potential novel viruses that could be detected with this method. Therefore, we considered the implementation of metadata is a lower prioritized task in this study. However, we plan to construct a web-based database which will provide the information of CRISPR spacers and discovered targeted genomes along with the predicted targeted host. In such a platform, the standardized MIUViG-based metadata will be certainly useful for searching and therefore considered as the highly prioritized task. Although we do not provide an MIUViG metadata at present, the provided supplementary data and descriptions include information about sequence source, assembly software, tools used for extracting CRISPR targeted sequences, predicted capsid genes, gene annotation method and results, and predicted targeting host. These data partially fulfill the MIUViG checklist and could be useful for some readers who want to investigate the result further.

The extracted CRISPR targeted TR sequences were compared to the VirSorter prediction result (page 16, line 371). The VirSorter program predicted 730 Microviridae MCP coding sequences (95.3%), 916 HK97 fold capsid coding sequences (42.5%), none of Inoviridae major coat coding sequences, and 109 sequences without a detectable capsid as positively viral (category >= 6). The VirSorter program has a good agreement with the predicted Microviridae species in our analysis. On the other hand, the program identified about half of HK97 capsid coding sequences and none of Inoviridae MCP coding sequences as viral. The disagreement of prediction results for HK97 coding sequences could be explained by the high diversity of their gene contents. We found that, among 257 discovered TR sequences similar to crAssphage, only ten sequences were predicted positively viral (category 3 and 6). crAssphages are known to have a variety of genes beside the essential core genes even in a closely related lineage. This fact might be complicating the prediction mechanism of the program, leading to less confident prediction results. This hypothesis also explains the good agreement of prediction results of Microviridae species. These viruses have small genomes occupied with few essential genes those are conserved across distant lineages, therefore these viruses possess less diversity of gene contents which could yield a good prediction agreement. None of Inoviridae MCP coding TR sequences including previously characterized genomes were predicted positively viral by the program. As we demonstrated, none of these Inoviridae MCP coding TR sequences were listed in either viral or plasmid databases. Therefore, this prediction result could be explained by the lack of reference sequences making impossible to build a viral gene database for these species internally used by the program. This topic was added to the revised manuscript.

The majority of TR sequences with detectable capsids were previously characterized viral genomes. Substantial portions of the sequences without detectable capsid genes were encoding plasmid-related genes. However, we found that the remaining portion of unclassified sequences had notably lower coding ratios. We further investigated two representative sequences among them and found that one of them is likely an extrachromosomal element targeted by a genuine CRISPR-Cas system. Although we do not know if this element is viral or not at this point. The discovery might be of interest. Accordingly, these topics were added the the revised manuscript (page 9, line 186).

Major Comments

In general, the pipeline is fairly well described, but lacks necessary benchmarking. There are a number of unexplained decisions that also need clarification. For example, the 93% sequence identity threshold for masked CRISPR hits is a very permissive threshold to select, and I question what that rationale was. Typically, identical matches or 1-mismatch are allowed given the short sequence length of spacers and to avoid spurious hits. Additional details for why this threshold was selected and a benchmarking of true vs. false positives is needed to ensure the underlying quality of the data is robust.

We believe this comment is almost identical to the first question of reviewer 1 and refer to our reply above.

Our purpose of using CRISPR spacers was to discover viral genomes targeted by CRISPR. These CRISPR targeted genomes are not necessarily being targeted by CRISPR at present, but mutated descendants escaped from CRISPR targetings, which is also our interest. To capture such escaped mutants in this study, we chose to use looser criteria (93%; telerate two mismatches) than the widely used criteria (95%; telerate one mismatch) in the previous studies. While the looser criteria may increase the sensitivity to capture the escaped mutants, the assessment of the spacer hit specificity was desired as the reviewer pointed out. To evaluate the specificity of spacer hit in a given criteria, we required “control” sequences; sequences not containing a genuine protospacer. However, there is a fundamental difficulty to design such control sequences, due to the nature of CRISPR spacers. It is known that CRISPR spacers could be acquired from various genomic sources, including DNA/RNA virus, plasmid, transposon and chromosomal DNA. Furthermore, partial prophage genomes are regularly encoded in bacterial/archaeal genomes. Therefore, we cannot define a metagenomic sequence that could absolutely not be a source of CRISPR spacer acquisition, making us unable to design a control. To complement this, we applied a graph-based clustering method to extract the regions with co-localized protospacers (S2 Fig), and evaluated the spacer hits by chance in a given criteria (Fig 5).

While the genuine protospacers from a viral genome are expected to co-localize together in a relatively small region, the false positive protospacers are expected to be scattered across the metagenome contigs almost randomly. We sought to extract the sequences with co-localized protospacers by using a graph-based clustering method (page 7, line 147). The aligned spacers were clustered based on the co-occurrence of protospacers. The clusters with less tightly connected (global clustering coefficient < 0.5), or small sizes (< 10) were rejected. 12,749 clusters comprising 591,189 spacers remained. These spacers are confidently co-aligned together with other spacers within the local regions. We considered a region containing at least 30% of a cluster member a genuine CRISPR targeted region. Such sequences were extracted and further analyzed.

The spacer hits-by-chance evaluation is analogous to the E-value from the blast program. This measurement was assessed as follows. With 95% identity, 192,801 spacers were aligned to terminally redundant (TR) sequences and one spacer was aligned to scrambled sequences. With 93% identity, 251,467 spacer sequences were aligned to TR sequences and 14 spacers were aligned to scrambled sequences (Fig 5). Although we observed an increased number of hits to scrambled sequences with 93% identity, 14 spacers would be acceptable considering that 58,666 more spacers were identified. Accordingly, we believe that the increased number of hits with the 93% identity threshold is unlikely to be the result of chance but more likely by the sequence diversity of TR sequences driven by selection pressures, including CRISPR targeting.

Overall, we conclude that the extracted CRISPR targeted sequences are mostly composed of genuine protospacers. We sought to evaluate the CRISPR avoidance phenomenon of viral genomes by using the discovered mutated genomes, which only briefly discussed in this manuscript (page 18, line 420 and page 22, line 516). We leave the further analysis of this topic in the future study.

The crAssphage DGR analysis and inference is poorly described and questionable. In particular, the link between the human gut crAssphage DGRs and the single (?) baboon sequence to infer function/activity is not grounded in much evidence. A more thorough analysis is warranted. Further, any inference of activity should be based on more than a single time point/sequence, and would ideally have longitudinal data for analysis of dynamics/function.

The DGRs in the crAssphages section were intended to show the discussion related to evolution using discovered genomes. We agree our approach is premature and lacks scientific proof. We decided to remove the entire section and leave it to our future study.

In general, the manuscript would benefit from significant revision to better organize the development/benchmarking of the pipeline and the subsequence analysis of the 11,000 gut metagenomes. For both, the authors should consider focusing on the lines of evidence to support their findings, instead of drawing conclusions based on little evidence. The authors should also thoroughly check for grammar/spelling.

Minor comments:

L. 54: Using the term “parasitic nature” does not accurately describe viral modes of activities, so I would suggest removing this phrase.

We agree that the word parasitic is not an appropriate word to describe viral activity. The sentence was removed.

L. 59: Metagenomics encompasses more than sequencing host-associated samples, so I encourage the authors to ensure an accurate definition. Further, the authors should also describe that beyond bulk metagenome sequencing from a given environment, laboratory-based approaches to target viral particles and subsequent sequencing (eg, viromics) is another widely used approach to sequence uncultivated viruses.

The sentence was changed to describe the metagenome more accurately.

We wrote about other viral genome sequencing approaches, such as filter-based virome sequencing, in an early draft. However, these references were removed because none of the methods directly relate to our approach.

L.75: A definition of “totally different” is warranted in this context. While reference-based virus detection tools do have limitations, many available tools rely on HMM-based searches which does enable recovery of divergent virus genomic sequences.

The phrase “totally different” does not provide a point of evolutionary perspective. We changed the phrase to explain more about the distant lineage of viral species.

**Have all data underlying the figures and results presented in the manuscript been provided?**
Large-scale datasets should be made available via a public repository as described in the *PLOS Computational Biology* [data availability policy](http://journals.plos.org/ploscompbiol/s/data-availability), and numerical data that underlies graphs or summary statistics should be provided in spreadsheet form as supporting information.

Reviewer #1: Yes

Reviewer #2: Yes

Reviewer #3: Yes

PLOS authors have the option to publish the peer review history of their article ([what does this mean?](https://journals.plos.org/ploscompbiol/s/editorial-and-peer-review-process#loc-peer-review-history)). If published, this will include your full peer review and any attached files.


If you choose “no”, your identity will remain anonymous but your review may still be made public.


**Do you want your identity to be public for this peer review?** For information about this choice, including consent withdrawal, please see our [Privacy Policy](https://www.plos.org/privacy-policy).

Reviewer #1: No

Reviewer #2: No

Reviewer #3: No

Figure Files:

While revising your submission, please upload your figure files to the Preflight Analysis and Conversion Engine (PACE) digital diagnostic tool, [https://pacev2.apexcovantage.com](https://pacev2.apexcovantage.com/). PACE helps ensure that figures meet PLOS requirements. To use PACE, you must first register as a user. Then, login and navigate to the UPLOAD tab, where you will find detailed instructions on how to use the tool. If you encounter any issues or have any questions when using PACE, please email us at [figures@plos.org](mailto:figures@plos.org).

Data Requirements:

Please note that, as a condition of publication, PLOS' data policy requires that you make available all data used to draw the conclusions outlined in your manuscript. Data must be deposited in an appropriate repository, included within the body of the manuscript, or uploaded as supporting information. This includes all numerical values that were used to generate graphs, histograms etc.. For an example in PLOS Biology see here: <http://www.plosbiology.org/article/info%3Adoi%2F10.1371%2Fjournal.pbio.1001908#s5>.

Reproducibility:

To enhance the reproducibility of your results, PLOS recommends that you deposit laboratory protocols in [protocols.io](http://protocols.io), where a protocol can be assigned its own identifier (DOI) such that it can be cited independently in the future. For instructions, please see [http://journals.plos.org/compbiol/s/submission-guidelines#loc-materials-and-methods](http://journals.plos.org/plospathogens/s/submission-guidelines)
