# Supplementary material for: Comprehensive discovery of CRISPR-targeted terminally redundant sequences in the human gut metagenome: Viruses, plasmids, and more
Source: PLoS Comput Biol. 2021 Oct 21;17(10):e1009428. doi: 10.1371/journal.pcbi.1009428 (PMC8530359; doi:10.1371/journal.pcbi.1009428)
Supplement: Attachment — Submitted filename: Response_to_reviewers.docx [file pcbi.1009428.s013.docx]

**Response to the reviewers:**

**Please note here if the review is uploaded as an attachment.**

Reviewer #1: The authors have addressed three of my major comments adequately. However, the most important issue, namely, comment #4, on the lack of an appropriate characterization of the identified virus genomes, remains unresolved. I think this type of analysis is a must in a paper on virus discovery.

We thank the reviewer for this valuable comment. To characterize a subset of the identified viral genomes, we performed a phylogenetic analysis of the discovered *Inoviridae* species and found that the discovered genomes formed a clade of lineages that were diverse from the RefSeq-recorded genomes. The sequence diversity of these clades satisfies the ICTV proposed condition of genus definition (less than 50% sequence identities in Zot protein homologues). This result was added to the revised manuscript (Pages 19, 27, and Figure 4).

Reviewer #2: I appreciate the authors’ genuine efforts to address the issues raised in my review, but I still find fundamental problems with the host taxonomy inference, which is key to the study. The authors attempted to address the concern I raised and added an ad-hoc (the results of which support the issues I’ve brought up). However, I find the host inference analysis *conceptually* problematic. The high rates of HGT, acknowledged by the authors, render the host inference ineffective when relying on an assembled reference database such as RefSeq. Due to the frequent HGT events, observing a directed repeat (DR) in a given organism within the reference database does not mean that the DR resides in the same host in the analyzed sample. Thus the host of a virus targeted by a spacer flanked with DRs cannot be reliably determined based on the presence of the DR in a specific host in RefSeq.

The authors try to deal with this to a certain extent by disregarding DRs that appear in RefSeq in multiple hosts. But this assumes that the representation of CRISPRs in various hosts in RefSeq is comprehensive and static. However, in actuality, it is partial and very much dynamic. Thus, the host inference analysis, which is a major part of this manuscript, is highly problematic in its current form.

We thank the reviewer for this in-depth discussion of our work. We understand and agree with the reviewer’s statement that host prediction based on DR-to-RefSeq is problematic because of our inability to characterize recent HGT, which could influence these results. Probably, the reviewer also agrees that there is limited data about how frequently HGT of CRISPRs occurs in the present populations. It is not possible to determine the extent of which the true CRISPR loci of a given species, at a specific time point, are represented in the RefSeq database until we obtain a comprehensive picture of the metagenome. Faced with these limitations, we still consider that the DR-to-RefSeq based host prediction has scientific merit as long as it is qualified and interpreted with appropriate caveats. To validate our targeting host prediction result using non-DR sequences that the reviewer may consider to be more indicative of a given taxa, we used tRNA genes encoded in TR sequences to infer the probable hosts, finding 93% agreement at the taxonomic level of order. Furthermore, we compared the targeting host prediction result with the result from recently published article. As shown in a recent publication from *Nature Microbiology* (Nayfach et al., 2021, https://doi.org/10.1038/s41564-021-00928-6), the authors extracted spacers from taxonomically assigned contigs and aligned these spacers to the predicted viral contigs, thus their result is appropriate to perform cross-study comparison. Using host-predicted sequences that appeared in both studies, we found that more than 95% of predicted hosts had agreement between the studies for all taxonomic levels at and above order. Thus, we conclude that, for human gut metagenomes, which have been deeply sequenced over the past decade, DR-to-RefSeq based targeting host prediction is consistent at higher taxonomic order and is unlikely to be highly problematic. In other environments which are more poorly sampled, the reviewer’s concern of the problems with targeting host inference are likely more valid, and we would avoid using DR-to-RefSeq based host prediction in such cases.

Moreover, we state that even if we use spacers extracted from taxonomically assigned contigs for the host prediction, the host predicted based on this method does not necessarily represent the host of a spacer-aligned viral genome. Viruses possibly undergo HGT and switch hosts, and there is no consensus regarding the frequency of these events. A spacer aligned to a viral genome is but a record of infection that occurred in the past and does not directly connect to the current infecting host.

These results and statements were added to the revised manuscript (Pages 12 and 26).

Currently, the spacer-based host prediction method is used in most virome studies and seems to be basically accepted widely. We seek to carefully investigate particularly the evolution of CRISPR loci, and rather than the disagreement regarding the host prediction results, we might find a sign of host-switching events. We leave this issue, and more precise descriptions could be provided in future studies.

Following my comment on the matter, the authors referred to plasmids and transposons in different parts of the manuscript. However, the title of the manuscript, as well as the abstract and major parts of the main text, still refer solely to viruses. This is misleading, as more than 59% of the small TR sequences and more than 15% of the large TR sequences detected in this study seem to originate from plasmids or similar elements.

We agree that the content of the manuscript does not completely match the title, abstract, and introduction. Although our initial motivation was to uncover viral genomes, a substantial portion of the results now pertains to nonviral sequences. Accordingly, we have altered the title, abstract, and introduction, to fit the results.

**Have the authors made all data and (if applicable) computational code underlying the findings in their manuscript fully available?**

The [PLOS Data policy](https://journals.plos.org/ploscompbiol/s/materials-and-software-sharing) requires authors to make all data and code underlying the findings described in their manuscript fully available without restriction, with rare exception (please refer to the Data Availability Statement in the manuscript PDF file). The data and code should be provided as part of the manuscript or its supporting information, or deposited to a public repository. For example, in addition to summary statistics, the data points behind means, medians and variance measures should be available. If there are restrictions on publicly sharing data or code —e.g. participant privacy or use of data from a third party—those must be specified.

Reviewer #1: Yes

Reviewer #2: Yes

PLOS authors have the option to publish the peer review history of their article ([what does this mean?](https://journals.plos.org/ploscompbiol/s/editorial-and-peer-review-process#loc-peer-review-history)). If published, this will include your full peer review and any attached files.

If you choose “no”, your identity will remain anonymous but your review may still be made public.

**Do you want your identity to be public for this peer review?** For information about this choice, including consent withdrawal, please see our [Privacy Policy](https://www.plos.org/privacy-policy).

Reviewer #1: No

Reviewer #2: No

Figure Files:

While revising your submission, please upload your figure files to the Preflight Analysis and Conversion Engine (PACE) digital diagnostic tool, [https://pacev2.apexcovantage.com](https://pacev2.apexcovantage.com/). PACE helps ensure that figures meet PLOS requirements. To use PACE, you must first register as a user. Then, login and navigate to the UPLOAD tab, where you will find detailed instructions on how to use the tool. If you encounter any issues or have any questions when using PACE, please email us at figures@plos.org.

Data Requirements:

Please note that, as a condition of publication, PLOS' data policy requires that you make available all data used to draw the conclusions outlined in your manuscript. Data must be deposited in an appropriate repository, included within the body of the manuscript, or uploaded as supporting information. This includes all numerical values that were used to generate graphs, histograms etc.. For an example in PLOS Biology see here: <http://www.plosbiology.org/article/info%3Adoi%2F10.1371%2Fjournal.pbio.1001908#s5>.

Reproducibility:

To enhance the reproducibility of your results, we recommend that you deposit your laboratory protocols in [protocols.io](http://protocols.io/), where a protocol can be assigned its own identifier (DOI) such that it can be cited independently in the future. Additionally, PLOS ONE offers an option to publish peer-reviewed clinical study protocols. Read more information on sharing protocols at <https://plos.org/protocols?utm_medium=editorial-email&utm_source=authorletters&utm_campaign=protocols>
